# Supplementary material for: A Likelihood Approach for Real-Time Calibration of Stochastic Compartmental Epidemic Models
Source: PLoS Comput Biol. 2017 Jan 17;13(1):e1005257. doi: 10.1371/journal.pcbi.1005257 (PMC5240920; doi:10.1371/journal.pcbi.1005257)
Supplement: S1 File — (TAR.GZ) [file pcbi.1005257.s014.tar.gz › HSPH_Online-SI-Revision/MSS10/n1-severe/n1-severe_NEW2_table0.pdf]

1	newly 3 weeks spec	27.	17.	{0., 37.}	56.	0.585449	newly 3 weeks cumul	92.	86.59	{23., 160.}	43.8804	0.259726	newly 5 weeks spec	28.	5.43	{0., 17.}	81.25	1.52524	newly 5 weeks cumul	149.	101.85	{23., 188.}	43.4161	0.321377	newly 54 weeks cumul	407.	109.32	{23., 212.}	73.14	0.690593
2	newly 3 weeks spec	51.	27.44	{5., 50.}	50.902	0.494397	newly 3 weeks cumul	151.	121.74	{45., 202.}	35.6424	0.23369	newly 5 weeks spec	43.	9.89	{0., 23.}	77.0465	1.05212	newly 5 weeks cumul	237.	148.09	{54., 255.}	41.481	0.305294	newly 56 weeks cumul	398.	163.72	{56., 271.}	58.8643	0.474361
3	newly 3 weeks spec	41.	25.18	{1., 48.}	49.2195	0.540864	newly 3 weeks cumul	126.	108.58	{28., 202.}	41.9683	0.267024	newly 5 weeks spec	34.	10.04	{0., 28.}	71.8235	1.16402	newly 5 weeks cumul	205.	135.14	{37., 235.}	42.8683	0.330843	newly 55 weeks cumul	432.	151.61	{37., 278.}	64.9051	0.586478
4	newly 3 weeks spec	70.	77.93	{66., 91.}	14.7571	0.059253	newly 3 weeks cumul	194.	241.08	{212., 263.}	24.3196	0.0930167	newly 5 weeks spec	40.	58.23	{46., 69.}	45.875	0.1592	newly 5 weeks cumul	270.	369.31	{335., 396.}	36.7963	0.134995	newly 58 weeks cumul	393.	591.84	{530., 630.}	50.5954	0.17643
5	newly 3 weeks spec	50.	58.57	{42., 74.}	24.22	0.0951658	newly 3 weeks cumul	135.	194.33	{149., 237.}	45.0148	0.156424	newly 5 weeks spec	19.	41.81	{30., 54.}	121.	0.332583	newly 5 weeks cumul	204.	285.94	{230., 338.}	41.7157	0.148304	newly 57 weeks cumul	333.	421.95	{350., 505.}	30.4354	0.116115
6	newly 3 weeks spec	37.	37.18	{26., 47.}	18.1622	0.0815389	newly 3 weeks cumul	122.	107.6	{85., 126.}	14.6557	0.0723371	newly 5 weeks spec	35.	37.48	{26., 48.}	21.2	0.0904183	newly 5 weeks cumul	196.	182.68	{147., 213.}	11.8163	0.0562942	newly 54 weeks cumul	501.	496.66	{381., 588.}	13.3932	0.0600782
7	newly 3 weeks spec	30.	38.08	{23., 52.}	39.2	0.177457	newly 3 weeks cumul	90.	121.32	{84., 149.}	42.	0.16387	newly 5 weeks spec	29.	32.43	{17., 46.}	33.8966	0.195356	newly 5 weeks cumul	156.	188.64	{123., 231.}	32.3333	0.14225	newly 54 weeks cumul	252.	362.43	{208., 457.}	55.0278	0.21834
8	newly 3 weeks spec	38.	76.78	{65., 88.}	102.737	0.304332	newly 3 weeks cumul	207.	264.76	{243., 287.}	28.087	0.10658	newly 5 weeks spec	30.	49.29	{39., 60.}	66.1667	0.22086	newly 5 weeks cumul	276.	377.33	{348., 404.}	37.2428	0.137137	newly 58 weeks cumul	301.	538.58	{471., 586.}	79.5681	0.253003
9	newly 3 weeks spec	23.	43.9	{35., 53.}	90.8696	0.274811	newly 3 weeks cumul	75.	128.94	{113., 143.}	71.92	0.233319	newly 5 weeks spec	38.	42.22	{35., 50.}	15.5263	0.0620945	newly 5 weeks cumul	137.	216.4	{191., 240.}	57.9562	0.196869	newly 54 weeks cumul	335.	546.53	{504., 579.}	63.1433	0.211875
10	newly 3 weeks spec	42.	59.84	{41., 77.}	45.2381	0.15449	newly 3 weeks cumul	146.	206.63	{157., 248.}	41.8014	0.145435	newly 5 weeks spec	28.	38.99	{24., 53.}	45.75	0.156656	newly 5 weeks cumul	205.	294.62	{221., 359.}	44.2146	0.151769	newly 54 weeks cumul	314.	418.89	{300., 523.}	36.4618	0.127655
11	newly 3 weeks spec	26.	43.88	{32., 55.}	68.8462	0.218354	newly 3 weeks cumul	86.	134.36	{110., 162.}	56.2326	0.189069	newly 5 weeks spec	24.	35.69	{22., 48.}	51.625	0.169451	newly 5 weeks cumul	140.	208.57	{167., 248.}	48.9786	0.168683	newly 51 weeks cumul	390.	378.32	{314., 430.}	9.17436	0.0414856
12	newly 3 weeks spec	37.	72.18	{55., 88.}	95.0811	0.283651	newly 3 weeks cumul	137.	262.06	{223., 290.}	91.2847	0.279651	newly 5 weeks spec	21.	44.3	{29., 55.}	111.429	0.313889	newly 5 weeks cumul	184.	365.1	{300., 395.}	98.4239	0.29526	newly 59 weeks cumul	237.	496.1	{362., 540.}	109.325	0.31563
13	newly 3 weeks spec	52.	37.19	{17., 56.}	32.4808	0.212913	newly 3 weeks cumul	157.	125.44	{73., 171.}	24.1274	0.138582	newly 5 weeks spec	31.	24.04	{9., 42.}	37.4194	0.247658	newly 5 weeks cumul	230.	179.71	{107., 253.}	25.9	0.15169	newly 54 weeks cumul	413.	246.58	{161., 323.}	43.0847	0.268795
14	newly 3 weeks spec	54.	83.08	{66., 98.}	57.7407	0.199589	newly 3 weeks cumul	204.	252.35	{230., 290.}	28.3775	0.112473	newly 5 weeks spec	35.	60.29	{47., 74.}	81.6857	0.30061	newly 5 weeks cumul	282.	385.41	{348., 428.}	41.9823	0.158227	newly 59 weeks cumul	417.	609.	{577., 663.}	53.1942	0.19691
15	newly 3 weeks spec	15.	42.27	{31., 53.}	181.8	0.441289	newly 3 weeks cumul	83.	147.65	{126., 168.}	77.8916	0.247178	newly 5 weeks spec	28.	29.47	{20., 40.}	24.8929	0.107882	newly 5 weeks cumul	134.	212.68	{174., 242.}	58.8806	0.197911	newly 53 weeks cumul	180.	321.81	{245., 373.}	78.7833	0.24739
16	newly 3 weeks spec	53.	60.65	{42., 79.}	27.2642	0.114774	newly 3 weeks cumul	165.	186.71	{141., 229.}	21.2667	0.0890836	newly 5 weeks spec	40.	47.77	{28., 63.}	35.225	0.145789	newly 5 weeks cumul	245.	289.05	{206., 355.}	25.9224	0.105475	newly 59 weeks cumul	406.	453.54	{297., 606.}	27.8867	0.118058
17	newly 3 weeks spec	52.	42.48	{12., 73.}	40.3077	0.259273	newly 3 weeks cumul	182.	179.39	{86., 244.}	26.1374	0.128971	newly 5 weeks spec	22.	23.87	{1., 53.}	86.8636	0.594576	newly 5 weeks cumul	246.	236.33	{108., 359.}	34.5894	0.172001	newly 59 weeks cumul	311.	304.17	{122., 541.}	50.0804	0.245533
18	newly 3 weeks spec	25.	55.65	{41., 72.}	122.76	0.336474	newly 3 weeks cumul	80.	151.21	{113., 187.}	89.0375	0.268348	newly 5 weeks spec	24.	54.8	{37., 69.}	128.333	0.348306	newly 5 weeks cumul	126.	263.36	{200., 317.}	109.016	0.3132	newly 59 weeks cumul	490.	591.	{522., 662.}	21.9755	0.0853582
19	newly 3 weeks spec	33.	42.65	{33., 53.}	33.1818	0.12343	newly 3 weeks cumul	111.	145.22	{130., 163.}	32.2523	0.121312	newly 5 weeks spec	35.	32.5	{23., 42.}	18.8	0.0938299	newly 5 weeks cumul	180.	212.96	{193., 239.}	20.2333	0.0808197	newly 52 weeks cumul	331.	349.33	{313., 387.}	10.5106	0.047105
20	newly 3 weeks spec	32.	57.15	{47., 68.}	78.5938	0.246946	newly 3 weeks cumul	124.	198.31	{181., 213.}	59.9274	0.203106	newly 5 weeks spec	27.	42.73	{33., 52.}	58.3333	0.193502	newly 5 weeks cumul	188.	290.88	{271., 307.}	54.7234	0.189052	newly 52 weeks cumul	258.	457.28	{433., 476.}	77.2403	0.248263
21	newly 3 weeks spec	34.	31.52	{3., 56.}	45.4118	0.355578	newly 3 weeks cumul	127.	116.33	{47., 188.}	34.7323	0.198982	newly 5 weeks spec	35.	18.53	{0., 35.}	53.6286	0.638728	newly 5 weeks cumul	201.	160.25	{58., 262.}	34.4428	0.22894	newly 59 weeks cumul	389.	201.94	{60., 320.}	49.4344	0.382611
22	newly 3 weeks spec	28.	20.63	{4., 35.}	41.75	0.30052	newly 3 weeks cumul	132.	119.84	{66., 166.}	24.9545	0.132217	newly 5 weeks spec	29.	5.73	{0., 14.}	80.2414	1.10106	newly 5 weeks cumul	187.	137.42	{72., 193.}	29.2193	0.176132	newly 53 weeks cumul	239.	143.07	{72., 198.}	40.1381	0.25568
23	newly 3 weeks spec	28.	17.64	{0., 40.}	57.3571	0.675579	newly 3 weeks cumul	106.	84.87	{12., 157.}	42.5755	0.307031	newly 5 weeks spec	31.	6.06	{0., 19.}	81.0968	1.42628	newly 5 weeks cumul	167.	101.48	{12., 184.}	46.2156	0.387966	newly 55 weeks cumul	324.	108.47	{12., 200.}	66.5216	0.627701
24	newly 3 weeks spec	36.	14.54	{0., 32.}	63.4444	0.814376	newly 3 weeks cumul	101.	74.09	{19., 129.}	41.7129	0.281226	newly 5 weeks spec	23.	4.	{0., 12.}	83.2174	1.47405	newly 5 weeks cumul	158.	86.35	{19., 155.}	48.7278	0.379713	newly 52 weeks cumul	238.	93.2	{19., 174.}	60.8403	0.521153
25	newly 3 weeks spec	53.	21.61	{7., 36.}	59.2264	0.462954	newly 3 weeks cumul	164.	108.9	{59., 157.}	34.2561	0.210353	newly 5 weeks spec	31.	7.32	{0., 15.}	76.3871	0.978255	newly 5 weeks cumul	226.	129.47	{70., 183.}	42.7124	0.271957	newly 50 weeks cumul	295.	138.62	{70., 197.}	53.0102	0.360203
26	newly 3 weeks spec	42.	21.12	{1., 43.}	53.6667	0.579476	newly 3 weeks cumul	125.	95.45	{32., 157.}	36.696	0.239681	newly 5 weeks spec	18.	10.23	{0., 27.}	68.5	0.871045	newly 5 weeks cumul	181.	121.23	{32., 214.}	41.3646	0.296035	newly 57 weeks cumul	332.	146.54	{32., 256.}	59.6867	0.48956
27	newly 3 weeks spec	52.	32.82	{5., 54.}	41.4615	0.42308	newly 3 weeks cumul	169.	159.01	{85., 221.}	24.3728	0.133461	newly 5 weeks spec	14.	14.96	{0., 36.}	81.1429	0.664224	newly 5 weeks cumul	215.	194.97	{86., 277.}	26.4326	0.149953	newly 56 weeks cumul	229.	227.51	{108., 383.}	34.5633	0.174603
28	newly 3 weeks spec	51.	76.64	{63., 91.}	50.3529	0.172548	newly 3 weeks cumul	140.	222.91	{199., 248.}	59.3071	0.200138	newly 5 weeks spec	48.	64.4	{54., 75.}	34.3333	0.125118	newly 5 weeks cumul	238.	359.58	{330., 387.}	51.084	0.178248	newly 59 weeks cumul	446.	649.46	{616., 683.}	45.6188	0.162849
29	newly 3 weeks spec	34.	37.53	{17., 54.}	36.9118	0.161713	newly 3 weeks cumul	114.	119.89	{72., 163.}	29.5351	0.129004	newly 5 weeks spec	17.	27.86	{11., 45.}	80.4706	0.259227	newly 5 weeks cumul	151.	181.5	{106., 250.}	37.2185	0.144045	newly 52 weeks cumul	284.	320.96	{158., 475.}	44.2606	0.188906
30	newly 3 weeks spec	37.	18.89	{7., 32.}	50.1892	0.364801	newly 3 weeks cumul	120.	77.36	{45., 113.}	36.4667	0.219708	newly 5 weeks spec	28.	8.88	{1., 17.}	68.2857	0.693181	newly 5 weeks cumul	177.	99.11	{56., 143.}	44.1186	0.279891	newly 53 weeks cumul	261.	115.59	{63., 168.}	55.7126	0.383806
31	newly 3 weeks spec	31.	39.57	{29., 51.}	31.7742	0.117379	newly 3 weeks cumul	87.	120.15	{101., 137.}	39.4138	0.142414	newly 5 weeks spec	16.	36.05	{24., 46.}	125.938	0.341703	newly 5 weeks cumul	126.	194.96	{170., 219.}	55.7937	0.190432	newly 54 weeks cumul	328.	462.83	{369., 521.}	42.4055	0.151265
32	newly 3 weeks spec	17.	54.03	{41., 68.}	217.824	0.494475	newly 3 weeks cumul	59.	151.52	{127., 178.}	156.814	0.405632	newly 5 weeks spec	26.	55.12	{43., 68.}	112.	0.319769	newly 5 weeks cumul	103.	261.56	{226., 304.}	153.942	0.401633	newly 56 weeks cumul	357.	588.53	{537., 648.}	64.8543	0.215781
33	newly 3 weeks spec	51.	63.16	{38., 84.}	35.9216	0.139733	newly 3 weeks cumul	172.	213.09	{150., 267.}	29.9593	0.114203	newly 5 weeks spec	50.	43.07	{21., 59.}	24.58	0.153471	newly 5 weeks cumul	284.	311.29	{201., 388.}	23.6232	0.101459	newly 59 weeks cumul	418.	441.04	{253., 556.}	26.9761	0.12141
34	newly 3 weeks spec	13.	24.17	{16., 33.}	86.6923	0.258619	newly 3 weeks cumul	56.	82.11	{67., 96.}	46.625	0.162182	newly 5 weeks spec	29.	19.95	{12., 28.}	32.4483	0.188372	newly 5 weeks cumul	105.	124.54	{106., 147.}	20.3048	0.0782155	newly 51 weeks cumul	255.	236.09	{188., 282.}	13.251	0.0630341
35	newly 3 weeks spec	56.	65.99	{50., 79.}	23.5893	0.0931768	newly 3 weeks cumul	196.	224.36	{190., 253.}	17.4694	0.0703084	newly 5 weeks spec	34.	44.63	{29., 57.}	38.1471	0.137976	newly 5 weeks cumul	269.	326.16	{265., 365.}	23.7175	0.0923462	newly 59 weeks cumul	333.	464.32	{340., 562.}	41.4294	0.145697
36	newly 3 weeks spec	35.	2.95	{0., 9.}	91.5714	1.68421	newly 3 weeks cumul	137.	79.72	{24., 134.}	43.2409	0.338645	newly 5 weeks spec	9.	0.07	{0., 0.}	99.2222	1.93579	newly 5 weeks cumul	166.	80.45	{24., 135.}	51.5361	0.413105	newly 60 weeks cumul	168.	80.45	{24., 135.}	52.1131	0.418306
37	newly 3 weeks spec	30.	18.05	{7., 28.}	42.6333	0.285385	newly 3 weeks cumul	103.	69.47	{40., 101.}	36.068	0.2098	newly 5 weeks spec	13.	10.62	{2., 19.}	42.	0.310059	newly 5 weeks cumul	136.	93.63	{53., 131.}	33.7868	0.198185	newly 50 weeks cumul	294.	122.52	{58., 174.}	58.3265	0.411074
38	newly 3 weeks spec	36.	58.63	{41., 75.}	64.1389	0.206146	newly 3 weeks cumul	146.	210.09	{172., 245.}	44.1301	0.15505	newly 5 weeks spec	22.	35.08	{21., 49.}	62.9091	0.201004	newly 5 weeks cumul	199.	291.02	{237., 336.}	46.5427	0.162087	newly 58 weeks cumul	228.	387.29	{298., 457.}	70.0307	0.22452
39	newly 3 weeks spec	27.	52.63	{41., 65.}	96.2593	0.286651	newly 3 weeks cumul	108.	167.29	{147., 187.}	54.8981	0.187911	newly 5 weeks spec	28.	43.75	{36., 53.}	60.25	0.215786	newly 5 weeks cumul	158.	260.41	{237., 290.}	65.1709	0.215852	newly 54 weeks cumul	281.	502.17	{471., 545.}	80.8292	0.258247
40	newly 3 weeks spec	44.	15.57	{0., 34.}	65.8409	0.784073	newly 3 weeks cumul	123.	87.82	{33., 142.}	38.5203	0.262452	newly 5 weeks spec	23.	3.7	{0., 12.}	84.	1.49768	newly 5 weeks cumul	179.	99.66	{34., 157.}	46.6704	0.349594	newly 60 weeks cumul	274.	102.84	{34., 165.}	62.5255	0.51522
41	newly 3 weeks spec	39.	66.21	{53., 78.}	69.7692	0.225064	newly 3 weeks cumul	166.	232.22	{204., 260.}	39.8916	0.143878	newly 5 weeks spec	19.	41.39	{31., 51.}	117.842	0.329271	newly 5 weeks cumul	210.	326.09	{283., 368.}	55.281	0.189248	newly 57 weeks cumul	257.	449.73	{389., 522.}	74.9922	0.240381
42	newly 3 weeks spec	38.	43.12	{34., 53.}	20.	0.0798298	newly 3 weeks cumul	107.	130.48	{107., 152.}	23.9252	0.0913422	newly 5 weeks spec	31.	36.26	{24., 46.}	28.9032	0.117953	newly 5 weeks cumul	165.	207.19	{169., 239.}	27.9091	0.105399	newly 57 weeks cumul	391.	446.55	{341., 522.}	22.8875	0.0961376
43	newly 3 weeks spec	32.	38.68	{28., 48.}	25.1875	0.0950544	newly 3 weeks cumul	103.	124.63	{105., 146.}	21.6214	0.082344	newly 5 weeks spec	12.	29.63	{19., 38.}	147.25	0.377862	newly 5 weeks cumul	138.	188.06	{157., 213.}	36.3043	0.131292	newly 50 weeks cumul	246.	330.82	{275., 377.}	34.9837	0.127339
44	newly 3 weeks spec	38.	62.65	{44., 80.}	65.2368	0.208389	newly 3 weeks cumul	148.	201.86	{163., 235.}	36.5135	0.130735	newly 5 weeks spec	25.	45.98	{35., 58.}	84.72	0.257004	newly 5 weeks cumul	218.	301.97	{253., 356.}	38.6835	0.138257	newly 58 weeks cumul	302.	441.96	{365., 499.}	46.3841	0.162046
45	newly 3 weeks spec	39.	46.06	{30., 59.}	28.6154	0.111248	newly 3 weeks cumul	137.	155.73	{114., 188.}	21.219	0.0852646	newly 5 weeks spec	24.	34.07	{18., 53.}	54.4583	0.200044	newly 5 weeks cumul	197.	230.96	{163., 281.}	25.3807	0.100324	newly 55 weeks cumul	330.	366.63	{222., 466.}	28.0152	0.119195
46	newly 3 weeks spec	24.	33.59	{21., 47.}	47.125	0.164423	newly 3 weeks cumul	77.	102.08	{75., 126.}	36.8312	0.133379	newly 5 weeks spec	26.	32.2	{19., 44.}	37.0769	0.139565	newly 5 weeks cumul	128.	167.77	{125., 213.}	35.1172	0.128199	newly 54 weeks cumul	420.	360.45	{278., 440.}	17.2167	0.0918659
47	newly 3 weeks spec	53.	84.98	{72., 100.}	61.3962	0.205622	newly 3 weeks cumul	225.	296.18	{273., 328.}	32.2844	0.121049	newly 5 weeks spec	15.	51.24	{40., 61.}	241.6	0.527083	newly 5 weeks cumul	267.	414.69	{392., 442.}	55.7341	0.191927	newly 60 weeks cumul	272.	555.95	{533., 591.}	104.68	0.30966
48	newly 3 weeks spec	21.	29.19	{13., 48.}	57.0952	0.203618	newly 3 weeks cumul	100.	103.55	{57., 155.}	31.01	0.139664	newly 5 weeks spec	11.	20.36	{5., 37.}	108.	0.364495	newly 5 weeks cumul	125.	146.67	{78., 230.}	38.184	0.15237	newly 54 weeks cumul	216.	223.55	{94., 415.}	47.6528	0.217583
49	newly 3 weeks spec	40.	73.43	{60., 87.}	84.325	0.262136	newly 3 weeks cumul	144.	240.54	{223., 259.}	67.9306	0.224381	newly 5 weeks spec	31.	50.77	{42., 62.}	67.3226	0.229717	newly 5 weeks cumul	205.	353.05	{334., 377.}	73.3805	0.238768	newly 58 weeks cumul	301.	530.61	{506., 567.}	77.99	0.250792
50	newly 3 weeks spec	29.	52.68	{42., 64.}	84.1379	0.263188	newly 3 weeks cumul	126.	150.97	{130., 171.}	22.246	0.0885486	newly 5 weeks spec	18.	49.53	{36., 62.}	178.5	0.455454	newly 5 weeks cumul	161.	252.03	{218., 281.}	58.2919	0.198813	newly 56 weeks cumul	315.	567.43	{511., 631.}	83.927	0.267404
